# Physicians’ views on optimal use and payment system for telemedicine: a qualitative study

**DOI:** 10.1186/s12913-023-09314-w

**Published:** 2023-03-28

**Authors:** Sarah Raes, Lieven Annemans, Ruben Willems, Jeroen Trybou

**Affiliations:** Department of Public Health and Primary Care, Ghent University, Ghent, 9000 Belgium

**Keywords:** Telemedicine, Payment system, Reimbursement, Physicians’ views, Qualitative study

## Abstract

**Background:**

Telemedicine is already in use in daily practice, but appropriate reimbursement and physician payment is falling behind in many countries. One reason is the limited availability of research on the matter. This research therefore examined physicians’ views on the optimal use and payment modalities for telemedicine.

**Methods:**

Sixty-one semi-structured interviews were conducted with physicians from 19 medical disciplines. Interviews were encoded using thematic analysis.

**Results:**

Telephone and video televisits tend not to be used as a first patient contact, except for triage of patients in urgency situations. Several minimum required modalities for the payment system of televisits and telemonitoring were identified. For televisits these were: (i) remuneration of both telephone- and videovisits to increase healthcare equity, (ii) little or no differentiation between videovisit and in-person visit fee to make videovisits financially attractive and sustainable for physicians, (iii) differentiation of televisit fee per medical discipline, and (iv) quality requirements such as mandatory reporting in the patient’s medical file. The identified minimum required modalities for telemonitoring were: (i) an alternative payment scheme than fee-for-service, (ii) remunerating not only physicians but also other involved health professionals, (iii) designating and remunerating a coordinator, and (iv) distinguishing sporadic vs. continuously follow-up.

**Conclusions:**

This research investigated the telemedicine usage behavior of physicians. Moreover, several minimum required modalities were identified for a physician-supported payment system of telemedicine, as these innovations necessitate challenging and innovation of the healthcare payment systems as well.

## Background

As the coronavirus disease 2019 (COVID-19) spread across the world in 2020, hospitals and physicians strongly reduced in-person patient visits and transitioned to telemedicine encounters [1]. Telemedicine is the delivery of healthcare and the exchange of healthcare information across distances [2]. Many forms of telemedicine have been developed, including televisits and telemonitoring. Televisits are alternatives to in-office patient visits. They were initially used for brief follow-up visits or visits with limited physical examination [3]. A study with neurologists indicated that televisits tend to be suited more for follow-up than for new referrals [4]. Reasons to not use televisits for new referrals are for instance increased medicolegal exposure, inability to perform a physical examination, and weaker patient-physician relationship [5]. Furthermore, gender, age, academic degrees, and academic-based practice tend not to be significant predictors of televisit utilization among orthopedic surgeons [3]. However, a higher experience level was associated with a slower televisit adoption time [3].

Telemonitoring is the use of telecommunication and information technology to monitor the health status of a patient from a distance [6]. Telemonitoring is believed to solve challenges regarding the increasing prevalence of elderly patients with chronic conditions [7]. However, different barriers arise with its implementation in daily practice. Firstly, telemonitoring is associated with an increase in workload, while there is often a shortage of medical staff and a high time pressure in hospitals [8]. Secondly, there are medicolegal problems in terms of who is responsible, and when and how often the data needs to be checked [9]. Thirdly, there is often a lack of clarity about when patients can be candidate for telemonitoring [8], and there can also be a lack of adequate physician compensation and patient reimbursement [10].

Although telemedicine is already used in daily practice by physicians [11], it is unclear from literature in what context they use telemedicine applications [12]. Moreover, appropriate physician payment systems and reimbursement policies are still lacking in many countries [13]. In Belgium, telephone visits are only temporarily reimbursed, and the fee for a telephone visit is €20, which is slightly lower than the lowest in-person visit fee (€26.24 for an anesthetist visit), and much lower than the highest fee for an in-person visit (€62.12 for an oncologist visit) [13, 14]. The in-person visit fees are presented in Table 1. Appropriate physician payment is important, as the financial impact of telemedicine tends to drive telemedicine adoption [15]. This was certainly visible during the COVID-19 pandemic, as physical contact had to be reduced, and physicians required telemedicine to cover their expenses [1]. However, little is known about the optimal modalities for physician payment of telemedicine. Therefore, this qualitative study investigated the views of physicians regarding their telemedicine usage behavior, and the minimum required modalities for a telemedicine payment system. Physicians were interviewed during this study, as the research focuses on physician payment. Moreover, as existing telemedicine literature often focuses on one medical discipline [3–5], this research investigated opinions of physicians from 19 different medical disciplines.

**Table 1:** In-person visit fees

Medical specialism	Fee (€)
Anaesthesiology	26.24
Cardiology	40.14
Clinical hematology	62.12
Dermato-venereology	34.33
Endocrine-diabetology	62.12
Gastro-enterology	40.14
General medicine	26.78
Geriatrics	42.10
Internal medicine	47.41
Medical oncology	62.12
Neuropsychiatry	50.42
Otorhinolaryngology or physical medicine and revalidation	29.61
Paediatrically oncology and hematology	62.12
Paediatrics	42.10
Pneumology	42.10
Psychiatry	50.42
Rheumatology	60.20
Other medical specialists	26.78

## Methods

Little is currently known about physicians’ views on optimal use and payment modalities of telemedicine. As this field of study is newly emerging and is still in the exploration phase, a qualitative research design was deemed fit [16].

### Payment system in Belgium

To provide a better understanding of the results, it is important to understand the payment or reimbursement system in which the participants operate. Belgian physicians are generally remunerated through a national fee-for-service system, which are conventions and agreements between healthcare providers and sickness funds [17]. For ambulatory care, patients pay the full costs for the service and then obtain a reimbursement from the sickness fund for part of the expense [17]. The co-payment is the difference between the full cost and the reimbursement. The co-payment depends on the convention status of the physician. Physicians have the option to respect the national fees (so-called conventioned physicians) [18]. Physicians who do not accede to these conventions are non-conventioned physicians and can ask a higher fee to patients (i.e. higher co-payment for patients) [18]. Physicians working in university hospitals receive a salary, which are negotiated through collective agreements with the university hospitals [17]. These university physicians also have the option to commit to the convention.

### Participants

Interview invitations were sent by email to Belgian physicians on 1 July 2020. Interviewees were specialized in the following 19 medical disciplines: anesthesia, cardiology, dermatology, endocrinology, family medicine, gastroenterology, general internal medicine, geriatrics, hematology, neurology, oncology, orthopedics, otorhinolaryngology, pediatrics, physical medicine and rehabilitation, pneumology, psychiatry, rheumatology, surgery (cardiac-, digestive-, oncology-, pediatric-, and thoracic surgery). A purposeful sampling strategy was used to include at least three physicians per medical discipline, and participants were selected from a list of physicians on the website of the National Institute of Health and Disability Insurance [19]. An overview of the sample characteristics can be found in Table 2. Practice type was an important characteristic, as Belgian physicians working in private hospitals or practices are remunerated through fee-for-service, while physicians working in university hospitals are remunerated by salary. Moreover, the convention status indicated whether physicians agree with the national fee-for-service system (70% in our sample compared to 69% on a national level [20]). Sampling continued until data saturation was reached, which occurred when no new insights emerged. In total, one out of ten physicians answered our recruitment call (or 64 physicians). Three physicians dropped out before the interview. A total of 61 physicians were interviewed between 6 and 2020 and 29 October 2020. The median time that the interviews lasted was 21 min (interquartile range: 17 min).

**Table 2:** Sample characteristics

Number of participants	61
Females % (n)	21% (13)
Dutch speaking / French	37 / 24
Experience % (n)	
Experience ≤ 10 years	10% (6)
10y < Experience ≤ 20y	26% (16)
20y < Experience ≤ 30y	36% (22)
Experience > 30 years	28% (17)
Practice type % (n)*	
University hospital, Prof. rank	36% (22)
General hospital	59% (36)
Private practice	11% (7)
Agrees with convention % (n)	70% (43)
Region % (n)	
Flanders	54% (33)
Wallonia	23% (14)
Brussels	23% (14)

Note. *The sum of the percentages is more than 100%, because two physicians work in two types of practices

### Procedure

An interview guideline was developed in a team-based approach with experts in the fields, and pilot tested with two physicians (Table 3). After the first ten interviews, the interview guideline was modified to address relevant topics that had been raised. The questions were broad and mostly open-ended to stimulate the natural flow of the interview. The interviews were conducted by the first author (SR), who had experience in the field of health economics and healthcare financing, and who was involved in the development of the interview guideline. The first author recorded, transcribed, and coded the interviews, and sent the transcriptions to the interviewees for member checking, to explore the credibility of the results [21]. The interviews were conducted in French or Dutch, and results were translated in English.

**Table 3:** Interview guideline

Theme	Questions
***General***	• Which types of telemedicine do you already use?
***Televisits***	
Use	• Did you use televisits before COVID-19? • Which type of televisits do you use (telephone or video) and why? • For what purpose and why do you use televisits?
Physician payment	• What do you think of the current payment system for televisits in Belgium? • How can the current payment system be improved?
***Telemonitoring***	
Use	• Which type of telemonitoring is already used? • Which type of telemonitoring will potentially be initiated in your daily practice and why?
Physician payment	• How can telemonitoring be reimbursed and paid?

### Analysis

The software QRS NVivo release 1.5 was used to analyze the interviews. Thematic and inductive analysis was used to analyze the data, by following the step-by-step process described by Braun and Clarke [22]: (1) familiarizing with data, including data transcription, (2) generating initial codes, (3) searching for themes, (4) review of themes, (5) naming of themes, and (6) producing the report. The identified codes are presented in Table 4.

**Table 4:** Themes and codes

Theme	Subtheme	Codes	Description
Televisit	Use	Follow-up televisits	Televisits are only used for follow-up upon patients.
		Mail	Televisits are used through mail.
		Video	Televisits are used through video.
		Telephone	Televisits are used through telephone.
		Use before COVID	Televisits are used before COVID.
		First visit	Televisits are almost never used as first visit.
	Physician payment	Mail	Payment should exist for televisits through mail.
		Video	Payment should exist for televisits through video-conferencing software.
		Telephone	Payment should exist for televisits through telephone.
		Low payment	Current payment is too low in comparison with in-person visit fee.
		Payment depends on medical discipline	Payment should depend on medical discipline.
		Susceptibility to abuse	Current payment is susceptible for abuse.
Telemonitoring	Use	Apps	Telemonitoring through apps is used.
		Wearables	Telemonitoring through wearables is used.
		Devices	Telemonitoring through devices is used.
	Physician payment	Other healthcare professionals	Payment for other healthcare professionals should also exist.
		Coordinator	Payment for a coordinator should exist.
		Other system than fee-for-service	An other system than fee-for-service is needed.
		Difference sporadic vs. continuous	There is a difference between sporadic and continuous telemonitoring.

Because of the large number of participants, we reinforced the qualitative study by quantitative counts of the interviewees discussing a specific subject. In the Results section, we therefore used specific pronouns indicating the number of interviewees [23]. The specific pronouns can be found in Table 5.

**Table 5:** Percentages of interviewees discussing a topic and matching pronouns

Percentage of interviewees	Pronoun
% < 25	Few
25 ≤ % < 50	Some
50 ≤ % < 75	Several
% ≥ 75	Most
100%	All

Ethical approval was obtained from the ethical committee of Ghent University Hospital (Registration number: BC-07343). Informed consent was given by the interviewees before the interview, where they declared to participate and agreed with recording the interview.

## Results

Except for televisits and telemonitoring, the physicians did not use another type of telemedicine.

### Televisits

Most Belgian physicians have started using televisits during the COVID-19 crisis, as physical contact with patients was no longer possible (87%). Few physicians used televisits before COVID-19, and only to a limited extent (13%).

#### A) types

Most physicians used telephone visits (95%), often to discuss test results with little impact for the patient. Only few physicians had already done a videovisit (16%). Some physicians highlighted technical issues as reason for not using a videovisit (e.g. no camera available). However, the willingness to use a videovisit was high, as several physicians indicated the advantage of seeing the face expressions of the patient: *‘… During a videovisit you can see that the patient looks pale, limp, not well, and thus dehydrated. … You learn a lot from just one glance.’* (Oncologist 1 – General hospital).

#### B) use

Most physicians would not use televisits as the first patient contact (78%), since the probability of a physical examination is high: *‘The problem is that I can’t assess everything with telemedicine. …. A physician has 5 senses: to feel, to smell, to see, to hear, and intuition, and should use them all’* (Pneumologist 1 – General hospital). Most physicians also expressed concerns about missing indications if they are not able to physically see the patient first. Only in rare circumstances, some physicians use televisit as first patient contact, for instance to triage patients based on emergency: ‘*Before the COVID-19 crisis, I already used it [telephone visits] to define the urgency and the importance of the necessity of seeing the patient, in order to triage patients.*’ (Abdominal surgeon 1 – General hospital).

When interviewed physicians used televisits for follow-up, it was mostly used to discuss test results with a limited emotional impact for the patient: *‘… I would recommend using televisits for communicating test or scan results in certain situations. However, using televisits is not acceptable if I would say to the patient: “Call me and if it is cancer, come back for an in-person visit to discuss the diagnosis”.’* (Otolaryngologist 1 – General hospital). Moreover, one physician defined a televisit as follows: *‘A televisit is a follow-up visit if the complaints and the context of the patient do not change’*. (Physical medicine and revalidation specialist 2 – University hospital)

#### C) physician payment

Firstly, almost all physicians indicated that they would like to be remunerated for televisits (both though telephone and video), either because they think that the work should be validated, or because it would stimulate them to adopt televisits (95% of interviewees). Secondly, most physicians indicated that €20 was not enough for a televisit: *‘… The problem is the fee of €20. For some physicians this implied a profit, for others a clear loss. This is of course unsustainable. …’* (Neurologist 3 – University hospital). Thirdly, as the fee of an in-person visit depends on the medical discipline, some physicians also indicated that the fee for a televisit should also depend on the medical discipline.

Lastly, some physicians indicated that the fee is too susceptible to abuse. There are no quality requirements linked to the fee, allowing physicians to bill the fee for almost any case. However, one physician indicated that although quality requirements are needed, they should not be too strict: *‘… there are few requirements. It makes it possible to bill trivial situations: “How are you? Then I see you next week. … I think that quality requirements will be necessary. But if you compare with France, I wonder whether that’s feasible: you have to prove that the patient is known in your practice, has visited the practice at least two times in the past year, and has a chronic condition from a certain list. Checking all these boxes would take an equally amount of time as an in-person visit. This would create a barrier for using televisits.’* (Dermatologist 1 – General hospital) Therefore, many physicians were in favor to prove the quality of the televisit by simply writing the conclusion of the televisit in the medical file of the patient.

### Telemonitoring

#### A) types

The interviewees used telemonitoring through apps (13% of interviewees), wearables (3% of interviewees), cardiac implantable electronic devices (CIEDs) (2% of interviewees), and insulin pumps (5% of interviewees). *Apps* tend to be used in neurology, hematology, gastro-enterology, oncology, and (digestive) surgery. They are used to follow-up complaints during or after treatment or surgery, as patients can indicate the side-effects in the app. Physicians can have a more detailed overview of the experienced side-effects with apps. When the side-effects are severe, physicians can also intervene in time by for instance adjusting the treatment.

*Wearables* are used to a limited extent in physical medicine and revalidation, and the rheumatologists and neurologists mentioned they could be used in their discipline as well. They are often used to register the activity rate of the patient, for instance after a knee surgery. According to the interviewees, wearables motivate patients to do their exercises and are useful as a more objective source of information than a conversation, as some patients do not tell the physician everything.

Telemonitoring *CIED*-patients was often used (e.g. pacemaker or implantable cardioverter-defibrillator – ICD). The interviewed cardiologists indicated it does not only reduce the number of physician visits for a patient, but it also prevents ICD-patients from unwanted shocks, caused by for instance technical device issues.

Telemonitoring of diabetes patients with an *insulin pump* is used to check the insulin value. If the value is not good, the physician may call the patient and instruct to change the insulin dose. According to the endocrinologists, telemonitoring is good for therapy compliance of the patient. However, no endocrinologist continuously followed up on the patient. The physician only checks the parameters sporadically: either during a follow-up visit, or when the patient reports problems.

#### B) physician payment

Several minimum required modalities of a telemonitoring payment system were identified by the interviewees. Firstly, the interviewees mentioned that there is a need for reimbursing telemonitoring and compensating physicians and other caregivers. According to the interviewees, it is not feasible for physicians to continuously follow up on the patients themselves, because this would highly increase the workload. Therefore, interviewees delegated most of the telemonitoring to nurse-specialists, who follow-up alerts continuously, and consult the responsible physician when necessary. The interviewees indicated that both physicians and nurse-specialists should be compensated correctly. Secondly, when multiple caregivers follow up on patients, the following interviewee mentions that a coordinator is necessary: *‘For telemonitoring, someone should have a coordinating function: telemonitoring should be set up; the effort of the different caregivers should be registered, and it should be compensated as such. And not just with one fee. Hospitals where caregivers are compensated fee-for-service, that is problematic.’* (Neurologist 3, University hospital) Thirdly, as indicated by the latter interviewee, some physicians indicated a need of another payment system than fee-for-service.

Lastly, the interviewees indicated a difference between a continuously and sporadic follow-up. Many interviewees followed up on patients sporadically, by checking the parameters during the in-person visit with the patient. Only two interviewed cardiologists continuously followed up on patients with CIEDs. Most interviewees indicated several reasons not to continuously follow up on the patient, such as the need for an organizational change, and the lack of physician payment.

## Discussion

This study analyzed the telemedicine use among physicians and their opinions about the minimum required modalities for a telemedicine payment system. The results indicated that participants only use televisits and telemonitoring. Additionally, the participants mentioned that they use telephone visits more often than videovisits. Furthermore, the participants indicated that they almost never use televisits as the first contact with the patient, because of the inability of a physical examination and the fear to miss indications. The participants use televisits sometimes as first contact to define the urgency and necessity of seeing the patient. Accordingly, literature for instance suggested to use a televisit as first contact to fasten the diagnosis and treatment of melanoma and nonmelanoma skin cancers, as images and clinical information are transferred to the dermatologist for review [24]. Moreover, our study investigated which telemedicine applications were used and when. In addition to our study, previous research investigated the theory behind telemedicine acceptance and use, and indicated that its predictors are mainly usefulness, social influences, and attitude [25].

Several minimum required modalities for a televisit payment system were identified. Firstly, the interviewees preferred to be remunerated for both telephone and videovisits. The option to use both televisit types improves equity in healthcare as videovisits were shown by Eberly et al. to be less feasible for older patients and patients with a lower income [26]. In fact, an ethical framework is currently missing for telemedicine, as the technology is still in development [27, 28]. An ethical framework could help to address these ethical issues [27, 29]. Secondly, as the Belgian fee of an in-person visit differentiates per medical discipline, several physicians had the opinion that the televisit fee should depend on the medical discipline. The televisit fee would otherwise be profitable for some medical disciplines but not for others. Thirdly, some participants indicated that the fee is susceptible to abuse. Therefore, the participants suggested to implement quality requirements, such as writing the televisit conclusion in the patient’s medical file, which in turn can be controlled by the authorities.

Fourthly, literature indicated that videovisits appeared to be less used among female patients and patients who do not speak the official language [26]. If these patients use more in-person visits and the fee for in-person visits is higher than the videovisit fee, then these patients pay more than patients using videovisits (assuming that patient co-payment is proportional to the actual fee). Moreover, these patients also have to pay for the transport to the physician practice. Therefore, offering a lower fee for videovisits than in-person visits might put these patients financially at a disadvantage. Similarly, the participants of our study had the opinion that the televisit fee should not differentiate too much from the in-person visit fee, as using televisits would otherwise be financially less attractive and perhaps unsustainable for physicians. However, payment parity (e.g. televisit fees are equal to in-person visit fees) discussions tend to be complex. Opponents to parity argue that televisits should be reimbursed at a lower amount than in-person visits because of the potential cost-savings associated with it [30]. Advocates for parity argue that if televisit fees do not align with in-person visit fees, physicians will not be stimulated to use televisits and stay with in-person visits [30]. Potential healthcare cost-savings will then never be realized. Although the interviewed physicians favor parity, payers disfavor parity as they are wary that the increased accessibility and convenience of televisits will lead to increased costs for them [29].

The results suggested that telemonitoring appeared to be mostly applied to CIED-patients. Several minimum required modalities for a telemonitoring payment system were identified. Firstly, the results indicated that the interviewed physicians tend to diverge from the traditional fee-for-service system as payment model for telemonitoring. Some studies indicated that telemonitoring might reduce the number of follow-up visits for patients with for example CIEDs [31], while a fee-for-service system is known to have the potential to increase supplied-induced demand [32]. Thus, a fee-for-service payment model for telemonitoring CIED-patients would hamper the potential reduction of follow-up visits. An episodic payment model might be better suited for this type of telemonitoring [15, 33]. In an episodic payment, often referred to as bundled payment, accountable health professionals receive a lump sum for providing relevant medical services within a defined time period [34]. However, episodic payment systems would perhaps less stimulate quality, because health professionals are not stimulated to regularly review data transfers [15]. Therefore, introducing quality bonuses might maintain the quality of telemonitoring. On the other hand, an episodic payment can be an implicit incentive for quality since the longer the monthly payments will continue, the longer the physician can be following the patient, and the more time the physician can invest in health prevention and promotion [32]. Ongoing monitoring of telemedicine quality becomes increasingly important [35]. However, appropriate quality measures for telemedicine are lacking [36], and only specified for rare cases [35].

Secondly, some participants had the opinion that the payment system for telemonitoring should consider that telemonitoring is performed by multiple health professionals (e.g. physician and nurse-specialists), who each perform individual tasks and collaborate at crucial moments, for instance when problematic signals are transmitted. Thirdly, confusion might arise when multiple health professionals are able to review the remote data, risking lack of poor review of remote alerts and poorly acting upon alerts [15]. Therefore, a participant suggested that a coordinator should be designated who is responsible for regularly checking (‘follow-up’) and acting upon remote alerts (‘monitoring’), and the coordinator should be paid accordingly. Lastly, results indicated a difference between sporadic and continuous telemonitoring. A telemonitoring payment system could be adapted to this distinction.

The main limitation of this study is selection bias. The electronic invitation to participate was sent to a large variety of physicians, but only one out of ten answered our call. Furthermore, participants were included from a wide variety of medical disciplines. Although general conclusions can be drawn from taking into account all these disciplines, conclusions per medical discipline were difficult to make considering only three or four physicians per medical discipline were interviewed. Moreover, nurse-specialists were not interviewed. Their opinions could be useful for optimizing the payment system of telemonitoring because of their potentially central role in telemonitoring services. Future research on telemonitoring payment should therefore include other healthcare professionals, such as nurse-specialists. Furthermore, a second interviewer and analyst were missing in our study. Lastly, several of the interviewees’ answers and opinions are mainly applicable in fee-for-service healthcare systems, so cautiousness about transferability is advised. Nonetheless, physicians, patients, and policymakers from other countries may benefit from our conclusions, as payment systems for telemedicine are in development in many countries.

## Conclusions

Appropriate reimbursement of telemedicine is essential for telemedicine use, as the financial impact of telemedicine on physicians and hospitals will be driving telemedicine adoption. However, few healthcare payment systems are sufficiently adapted to this innovation, forcing healthcare providers to choose for in-person encounters. Therefore, this study investigated telemedicine use among physicians, and their views on the optimal physician payment modalities for televisits and telemonitoring. Findings suggested to improve healthcare equity by remunerating both telephone and videovisits, and to remunerate videovisits almost equally to in-person visits to make videovisits financially acceptable and sustainable for physicians. Moreover, results indicated to reduce the importance of fee-for-service as payment system for telemonitoring and transition to episodic payment systems, while ensuring quality with quality bonuses or requirements. Challenging the traditional payment system is necessary, as innovation in the healthcare system often requires innovation of the system.

## Data Availability

The datasets generated and/or analyzed during the current study are not publicly available due to privacy protection but are available from the corresponding author on reasonable request.

## List of abbreviations

COVID-19: Coronavirus disease 2019
CIED: Cardiac Implantable Electronic Devices
ICD: Implantable Cardioverter-Defibrillator
